# First Case of Infective Endocarditis With Streptococcus equinus in an Immunocompetent Patient in North America: A Case Report and Review of Literature

**DOI:** 10.7759/cureus.19473

**Published:** 2021-11-11

**Authors:** Dinesh Keerty, Abraham T Yacoub, Thu-Cuc Nguyen, Elizabeth Haynes, John Greene

**Affiliations:** 1 Internal Medicine, Moffitt Cancer Center, Tampa, USA; 2 Infectious Diseases, Moffitt Cancer Center, Tampa, USA

**Keywords:** bacteremia, streptococcus equinus, antibiotics, group d streptococcus, endocarditis

## Abstract

Infective endocarditis (IE) can be caused by bacterial or fungal infections invading the endocardial surface of the heart, such as its valves and chambers. *Staphylococcus* and *Streptococcus* species are mainly responsible for IE. *Streptococcus equinus* (*S. equinus*) has been rarely noted to cause IE. We present a case of a 69-year-old white male with a past medical history of severe aortic regurgitation, who during an elective aortic heart valve replacement surgery was noted to have multiple plaque-like vegetations at the base of the mitral valve that were positive for *S. equinus*. To date, there are only four cases of *S. equinus *endocarditis reported worldwide, with a high possibility of our case being the first reported in North America.

## Introduction

Infectious endocarditis (IE) is a serious infectious disease and carries a high risk of morbidity and mortality. IE can be classified as either community-acquired or hospital-acquired. Community-acquired IE develops in the absence of recent healthcare involvement while healthcare-associated IE refers to development in the context of a recent healthcare setting, with the onset of symptoms ≥48 hours after hospitalization [1]. Common risk factors include older age (>60 years), male gender, history of intravenous drug use, and other comorbidities. *Staphylococcus *spp. and *Streptococcus *spp. are mainly responsible for IE. Among the *Streptococcus *species, *Streptococci viridans *is the main cause of IE, with about 17% of cases [2]. *Streptococcus equinus* (*S. equinus*) has been rarely noted to cause IE [3]. We present a case of *S. equinus *endocarditis found at the base of the mitral valve during an aortic valve replacement.

## Case presentation

A 69-year-old white male with aortic regurgitation was complaining of worsening shortness of breath on exertion, epigastric fullness, weight loss, and malaise for the past six months. He has been medically managed for his aortic regurgitation by the cardiology team over the past eight years. Two months ago, he underwent a cardiac catheterization to evaluate the severity of the aortic regurgitation, which revealed severe 4+ aortic regurgitation, markedly enlarged left ventricle, and an ejection fraction of 55%. Therefore, he was admitted to the hospital for elective aortic valve replacement surgery.

His past medical history is significant for hypertension, benign prostatic hyperplasia, diverticulitis, gastritis, lower gastrointestinal bleeding, and atrial fibrillation. He also underwent an inguinal hernia repair and vasectomy in the distant past. In 2013, a colonoscopy revealed a 3-4 mm polyp in the region of the hepatic flexure, and gastroscopy revealed a polyp in the gastric region; both were resected.

A few months ago, he went on a Scandinavian tour, in which he stated that “there were horses on board” but denied contact with the animals. In the past month, he developed right lower extremity cellulitis, which was treated with a seven-day course of oral antibiotics.

In the hospital, he denied nausea, vomiting, diarrhea, chest pains, fever, chills, or sweats. His vital signs were unremarkable. Physical examination revealed a two over six early diastolic murmur, which was loudest in the third left intercostal space, with a regular rate and rhythm, and no rubs or gallops. The point of maximal impulse was not displaced. There were no signs of peripheral edema, splinter hemorrhages, Osler’s nodes, or conjunctival petechiae findings. Laboratory workup was within normal limits (Table 1).

**Table 1:** Laboratory workup results. Pertinent lab values of the patient.

	Patient’s result	Normal value
Cell counts		
White blood cells	7.8 k/uL	4–10.9 k/uL
Hemoglobin	12.3 g/dL	13.4–16.9 g/dL
Platelets	373 k/uL	143–382 k/uL
Chemistry		
Alkaline phosphatase	82 U/L	40–130 U/L
Aspartate aminotransferase	13 U/L	10–50 U/L
Alanine aminotransferase	27 U/L	0–40 U/L
C-reactive protein	<0.9 mg/dL	0.9–100 mg/dL
Creatinine	0.9 mg/dL	0.7–1.3 mg/dL

**Figure 1:**
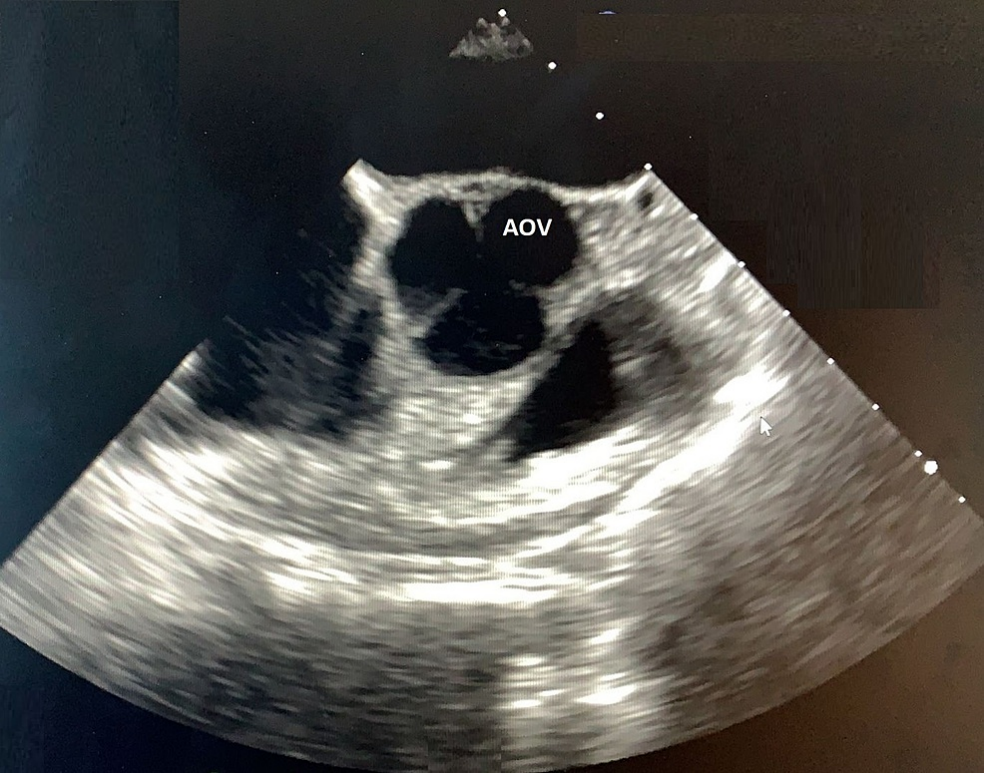
Transesophageal echocardiogram evaluating the aortic valve. AOV: aortic valve.

He underwent a transthoracic echocardiogram and a transesophageal echocardiogram for further evaluation of his aortic regurgitation (Figure 1).

There was no evidence of vegetation. He underwent an uneventful replacement of his aortic valve during his hospitalization. After replacing the diseased aortic valve with a bovine valve, multiple plaque-like vegetations were noticed at the base of the mitral valve just underneath the left coronary cuffs. The plaque-like vegetations were scraped and sent for cultures. Gram stain of the specimen showed gram-positive cocci in chains. He was treated with vancomycin 1 gram (g) intravenous (IV) every 12 hours, ceftriaxone 2 g IV daily, and metronidazole 500 mg IV every eight hours.

Eventually, the cultured vegetations grew *Streptococcus equinus*, which was susceptible to ceftriaxone. The specimen was also sent to the New York State Health Department where with the use of matrix-assisted laser desorption/ionization-time of flight (MALDI-TOF), *S. equinus* was confirmed.

Vancomycin and metronidazole were discontinued. The patient was discharged from the hospital with a course of ceftriaxone 2 g IV daily for six weeks based on the sensitivity report. Repeat blood cultures were negative for bacterial growth after six weeks. Because of the recent history of colonoscopy, it was not repeated. However, gastric endoscopy was performed, which revealed a recurrent polyp, which was removed.

## Discussion

*Streptococcus equinus* is a group D *streptococcus*, which is gram-positive, non-hemolytic, non-Enterococcus, bile-esculin positive, non-lactose fermenting, and non-mannitol fermenting microorganism. It is found predominantly in the alimentary tract of horses [4-5]. It has also been found in the alimentary tract of cows, humans, and food sources [5-7]. The 16S rDNA sequence of* Streptococcus gallolyticus* (*S. gallolyticus*), previously named* Streptococcus bovis* [8], differs from that of *S. equinus* by only 15 sites along with a 1455-base fragment [6]. These high degrees of similarity both in total DNA-DNA hybridization and in 16S rDNA sequence confirm that both *S. gallolyticus* and *S. equinus* belong to a single species [6]. Furthermore, these organisms do not grow in 6.5% salt and methylene-blue milk [9].

Patients with IE most commonly present with fever and chills, and weight loss, fatigue, myalgia, and arthralgia can also be present [10]. Congestive heart failure and cerebral emboli are the most common complications of IE [11]. The Duke criteria is a valuable tool for the diagnosis of IE [12]. Echocardiography is the method of choice for demonstrating endocardial involvement and transesophageal echocardiography (TEE) is more sensitive than transthoracic echocardiography (TTE) [13]. Broad-range polymerase chain reaction (PCR) amplification followed by direct sequencing is a reliable and accurate technology for pathogen detection and can be used as an adjunct to culture methods [14]. One major cause of negative blood cultures in patients with IE is receiving prior courses of antibiotics before blood cultures are obtained due to the suspicion of a non-cardiac bacterial infection [15]. In our case, the patient was admitted to the hospital one month before his aortic valve replacement therapy for right lower extremity cellulitis and was treated with a seven-day course of antibiotics. This prior antibiotic therapy may have led to negative blood cultures.

We speculate that the bacteria seeded the heart valve via hematogenous spread from the colon or the stomach. There is a strong association between group D *streptococcus*endocarditis such as *S. gallolyticus* and colorectal cancer [16]. *S. gallolyticus* endocarditis has developed in a patient with a colonic polyp [17]. Although the association between *S. equinus* endocarditis and colorectal cancer or a colonic polyp has never been documented due to its rarity, it is probable since both *S. gallolyticus* and *S. equinus* belong to the same species [6].

Our patient had undergone a colonoscopy in 2013, which revealed a colonic polyp in the region of the hepatic flexure. In addition, the patient had a recent gastroscopy, which revealed a polyp in the gastric region. The organism may have gained access to the bloodstream from the gastric polyp since *S. equinus* can tolerate a low pH environment [5]. The patient mentioned attending a Scandinavian tour in which horses were nearby. However, he had no contact with the horses as they both were segregated.

The standard treatment of group D *streptococcus *IE includes penicillin G 12-18 million units per day intravenously in four to six doses or daily ampicillin 100-200 mg/kg/day intravenously in four to six doses, or daily ceftriaxone 2 g/day intravenous [18]. A two-week treatment regimen includes penicillin G 12-18 million U/day intravenously in six doses or ampicillin 100-200 mg/kg/day intravenously in four to six doses or ceftriaxone 2 g/day intravenous in one dose with gentamicin 3 mg/kg/day intravenous in one dose or 4-5 mg/kg/day intravenously [18]. In beta-lactam allergic patients, alternative antibiotics include vancomycin 30 mg/kg/day intravenously in two doses [18]. Daptomycin can be used as second-line therapy after initial treatment failure [18].

To date, there are only four cases of *S. equinus* endocarditis reported worldwide [19-20]. We believe our case may be the first to be reported in North America.

## Conclusions

Infectious endocarditis is a clinical condition that requires timely attention by physicians because it carries a high risk of morbidity and mortality. IE can be classified as either community-acquired or hospital-acquired. Risk factors include age, gender, history of intravenous drug use, and other comorbidities. Patients can present with varying symptoms ranging from fever, fatigue, myalgias, or arthralgia. The Duke criteria is a valuable tool for the diagnosis of IE. Echocardiography is the method of choice for demonstrating endocardial involvement with transthoracic being superior to transesophageal. Infective endocarditis with *S. equinus* is rare but leads to high morbidity and mortality if left untreated. *S. equinus* could spread from a gastrointestinal source and pertinent gastric and colonic examinations are recommended. Standard treatment of group D *streptococcus* IE includes penicillin G and ampicillin, and ceftriaxone daptomycin can be used as second-line therapy after initial treatment failure. This infection should be recognized as an emerging zoonosis. Early detection is crucial and prolonged antibiotic therapy is indicated.
